# New paradigms in regenerative engineering: Emerging role of extracellular vesicles paired with instructive biomaterials

**DOI:** 10.32604/biocell.2022.018781

**Published:** 2022-02-07

**Authors:** W. Benton SWANSON, Yuji MISHINA

**Affiliations:** Department of Biologic and Materials Science & Prosthodontics, School of Dentistry, University of Michigan, Ann Arbor, MI, 48109, USA

**Keywords:** Biomaterials, Extracellular vesicles, Mesenchymal stem cells, Regenerative medicine, Tissue engineering

## Abstract

Mesenchymal stem cells (MSCs) have long been regarded as critical components of regenerative medicine strategies, given their multipotency and persistence in a variety of tissues. Recently, the specific role of MSCs in mediating regenerative outcomes has been attributed (in part) to secreted factors from transplanted cells, namely extracellular vesicles. This viewpoint manuscript highlights the promise of cell-derived extracellular vesicles as agents of regeneration, enhanced by synergy with appropriate biomaterials platforms. Extracellular vesicles are a potentially interesting regenerative tool to enhance the synergy between MSCs and biomaterials. As a result, we believe these technologies will improve patient outcomes through efficient therapeutic strategies resulting in predictable patient outcomes.

## Introduction

Originally conceived to solve the shortage of organs for transplantation, the field of tissue engineering has evolved to encompass a broad clinical scope including regeneration of simple and complex tissues in a variety of clinical settings (Langer and Vacanti, 1993). At their core, tissue engineering strategies rely on three tenants: isolated cells, inductive substances, and matrices to facilitate organization, largely biomaterials (Khademhosseini and Langer, 2016; Langer and Vacanti, 2016). Despite significant academic advances, clinical translation remains slow due to challenges concerning cell sourcing, manufacturing scale, standardization, and regulation (Hoffman et al., 2019).

Mesenchymal stromal cells (MSCs) have attracted significant attention as an ideal multipotent stem cell source since their discovery as fibroblast-colony forming cells (Friedenstein et al., 1970). MSCs are extracted from a variety of tissue sources and are capable of multilineage differentiation (Yingst and Hoffman, 1984). Over 800 clinical trials have been conducted to determine their therapeutic efficacy (Kabat et al., 2019; Squillaro et al., 2016). However, no MSC therapies have been formally approved for use in the United States Food and Drug Administration. Significant concerns around the large-scale preparation of MSCs remains challenging (Jayaraman et al., 2021; Phinney and Galipeau, 2019; Sensebé et al., 2013).

**FIGURE 1.**
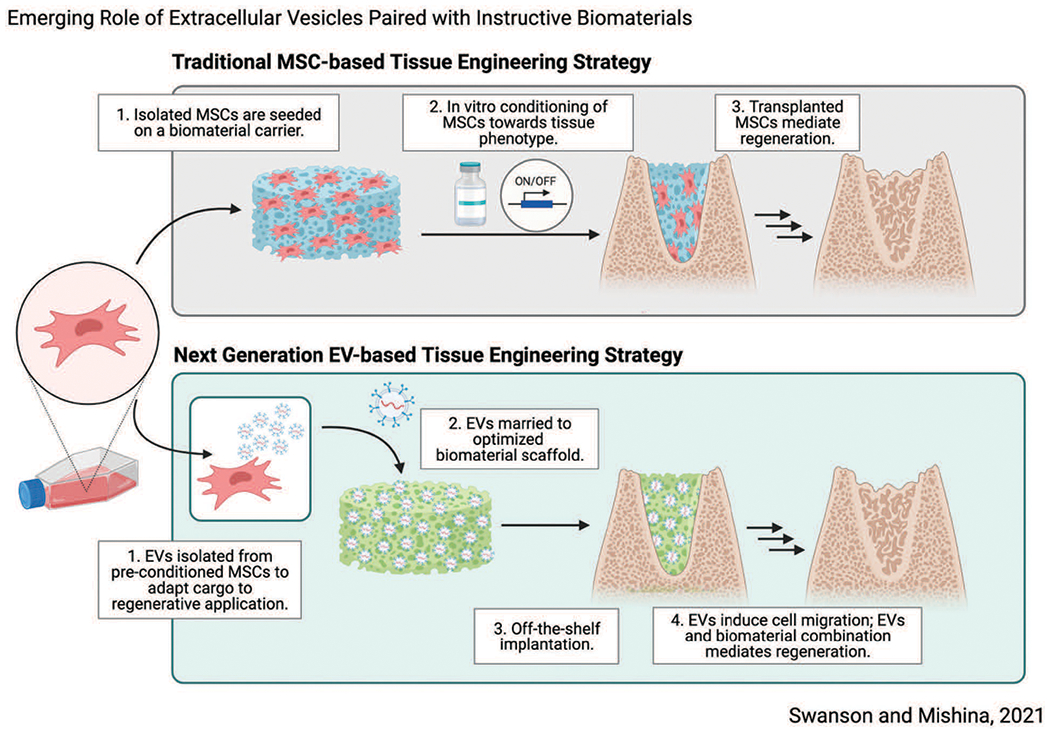
Schematic overview demonstrating next-generation tissue engineering therapeutic strategy which relies on synergy between biomaterial scaffolds and sustained release of EVs to induce tissue regeneration. Made with Biorender.

Concurrent with advances in tissue engineering, advances in molecular and developmental biology have significantly informed innovative tissue engineering strategies (Lenas, 2018). In this viewpoint we highlight recent advances in investigational therapeutics which pose significant translational advantages using extracellular vesicles as agents of regeneration, in novel combination with biomaterial platforms, illustrated in Fig. 1. We hypothesize that thoughtfully designed biomaterials paired with cell-instructive signals may induce predictable regeneration by endogenous cell sources, posing significant translational advantages as next generation tissue engineering therapeutics.

## Biomaterials Modulate Cell and Tissue Fate

MSCs respond to physical, chemical, and mechanical environment, providing a role for biomaterials-instructed regeneration (Jang and Kim, 2010; Leach and Whitehead, 2018). In addition to providing tissue organization in three dimensions, biomaterial features play a role in determining tissue fate through porosity (Loh and Choong, 2013), stiffness (Breuls et al., 2008), texture (Smith et al., 2009; Zhang and Ma, 2000), pore size (Gupte et al., 2018; Swanson et al., 2021), and chemical functionality (Zou et al., 2018), with the goal of replicating the niche or microenvironment of target cells and tissues to increase regenerative success (Williams, 2019). Biomaterials may be impregnated with growth factors or controlled release moieties to display inductive signals to cells, mimicking *in vitro* administration and secretion *in vivo*, which increases efficiency and minimizes off-target effects (Swanson et al., 2020b; Swanson et al., 2020c). Decellularized biomaterial matrices, containing residual proteins, are approved by the FDA in various forms and provide inspiration for a combination of inductive cue display within a biomaterial (Schmidt, 2012). Synthetic materials offer a greater degree of design tunability and manufacturing advantages (Agmon and Christman, 2016; Swanson and Ma, 2020); their fabrication methods are highly scalable, representing a clear path to clinical scale which is more cost-effective than cell-based therapies (Greenberg-Worisek et al., 2018; Sanz-Nogués and O’Brien, 2021; ten Ham et al., 2020).

## Secreted Factors Enhance Biomaterials-Based Regeneration

Kitami et al. (2016) demonstrate that prolonged survival of transplanted cells does not directly accelerate osseous wound healing, despite accelerated healing in defects treated with cells (Kitami et al., 2016). These results suggest that transplanted cells alone are not responsible for regenerative outcomes directly, yet they provide important instructive signals. Similar findings in transplanted adipose-derived MSCs have been reported (Muhammad et al., 2017). The secretome, the composite milieu of cells’ secreted factors which includes: proteins, growth factors, and extracellular vesicles (EVs), has recently been identified as a critical driver of cell fate (Pinho et al., 2020). Saha et al. (2019) demonstrated similar results in the functional recovery of ischemic myocardium after cardiac progenitor cell (CPC) transplantation, and specifically identified EVs produced by CPCs, one component of cellsecreted factors that is readily isolated, contained microRNAs associated with myocardial recovery. These findings suggest that transplanted cells may act as an *in-situ* drug factory, synthesizing inductive cues which catalyze regeneration, rather than directly participate (Moghadasi et al., 2021). In the context of tissue engineering, it is plausible to replace transplanted MSCs with secreted factors, such as EVs, in a way which mimics their natural secretion (Fig. 1).

## Growing Role for Extracellular Vesicles in Catalyzing Regeneration

EVs are lipid-bound vesicles with diameters in the range of 50–150 nm (Swanson et al., 2020a; Thery et al., 2018; Witwer et al., 2019). Originally thought to be a waste shedding mechanism by cells, recent evidence suggests that EVs are nature’s endogenous nanoparticle delivery system and a form of cell-cell communication, containing microRNAs and proteins (van Niel et al., 2018). Like stem cells, EVs have shown important therapeutic potentials in a variety of disease states and target tissues, outlined in Table 1.

EV-based therapeutics are promising regarding their translational and therapeutic potential. Ibrahim et al. (2014) isolated cardiosphere-derived cell EVs and profiled their molecular cargo to determine enriched miRNAs after demonstrating EV injection recapitulates the regenerative effects of transplanted cells. Inhibition of EV biosynthesis *in vivo* blocked these same effects. Interestingly, administration of the upregulated miR-146a reproduced only some, but not all, effects of EV administration. The authors propose EVs as a method of tying together regenerative paracrine and autocrine effects of cardiac progenitors without manually postulating their complex mixtures of signaling molecules.

The molecular cargo of EVs is reflective of its donor cell identity, and culture environment (Dai et al., 2019; Fevrier and Raposo, 2004; Quesenberry and Aliotta, 2010). This affords significant, large-scale cell culture manipulations to take place *in vitro* which tailor EV cargo towards specific regenerative applications, for example, by small molecule or growth factor treatment. It is also reasonable to consider biomaterial culture platforms as a method of large-scale EV manufacturing, given our understanding of biomaterial influences on cell phenotype. 3D cultures are also shown to increase EV yield in response to tissue-like organization (Lee et al., 2021; Rocha et al., 2019). Additionally, EVs isolated from highly controlled culture systems may be optimally tuned to educate naïve recipient cells (endogenous or transplanted) in recipient tissue defects, minimizing the requirement of preconditioned cells for transplantation.

Compared to MSCs, EVs exhibit “immune privilege” and demonstrate a better safety profile in terms of tumorigenicity and immunogenicity (Rani et al., 2015; Zhang et al., 2018b). EVs are shown to be well-tolerated without adverse immune responses or need for immunosuppressive agents (Mendt et al., 2018). EVs from immortalized cell lines represent an opportunity to standardize their biosynthesis and cargo (Deb et al., 2019; Kim et al., 2021; Swanson et al., 2020b) given that immortalized cells are less susceptible to change over time. Recombinant DNA technology may allow for further manipulation of the EV membrane or cargo, recently described as “designer exosomes” (Jafari et al., 2020). Recent literature suggests cross-species efficacy of EVs (Swanson et al., 2020b; Swanson et al., 2020c; Zhu et al., 2017); plant-derived EVs are also under investigation for various therapeutic uses (Akuma et al., 2019; Garaeva et al., 2021). As a result of recent interest in EV-based therapeutics, good manufacturing practices (GMP) have been developed for their commercial manufacturing (Bahr et al., 2020; Colao et al., 2018; Harn et al., 2020; Mendt et al., 2018).

The ideal regenerative therapeutic would allow for off-the-shelf clinical use and require minimal preparation, particularly for routine applications such as in clinical dentistry and dermatology. Researchers must consider that most healthcare settings do not have advanced tissue culture capability to handle or culture MSCs for use in tissue engineering applications, when required. Compared to MSCs, EVs are easily lyophilized and stored for future use (El Baradie et al., 2020; Swanson et al., 2020b). Charoenviriyakul et al. (2018) demonstrated that lyophilized EVs retained their activity for approximately 4 weeks even when stored at 25°C (room temperature), which poses significant clinical and commercial distribution advantages.

## Vision for Next-Generation Regenerative Technology

Despite numerous human clinical trials underway with EV-based therapeutics for a variety of clinical applications, most are limited to intravenous infusion or direct injection. EVs circulate the body rapidly, thereby requiring a high dose to reach therapeutic efficacy and pose risk for off-target effects. In the context of tissue engineering, the therapeutic effect is needed and desired locally. Our group and others have reported early developments in the delivery and sustained release of EVs by clinically and biologically relevant means. An important feature of these biomaterials platforms is that they are highly versatile. EV cargo may be changed (see Table 1 for examples) based on the clinical indication and desired outcomes, however the design of the platform technology remains otherwise unchanged. This allows for versatile and widespread use of these biomaterials technologies as platform technologies.

Hydrogels encapsulating EVs function to maintain EVs at the site of implantation, increasing their half-life *in vivo* (Zhang et al., 2018a). Historically hydrogels have had mixed success with the long-term encapsulation of cells due to mass transfer limitations. Because EVs are non-living, many fewer parameters must be considered. Gingival MSC in chitosan/silk hydrogel sponge accelerates wound healing on skin defects in diabetic mice by inducing neoepithelialization and angiogenesis to a greater degree than the hydrogel alone (Shi et al., 2017). Other examples of hydrogel-based EV delivery are discussed by Riau et al. (2019).

Synthetic biodegradable materials which encapsulate EVs in controlled amounts allow for their controlled dosing and long-term sustained release. We demonstrated the first report of an EV-containing poly(lactic acid-co-glycolic acid) (PLGA) microsphere. Over time, the PLGA polymer is degraded to allow EV release to local cells. We demonstrated that this delivery system was sufficient to induce odontogenesis (mineralized dentin formation) as a novel pulp-capping strategy to protect vital tooth tissue, where EV or cell administration would be otherwise limited. In this way, EVs are locally released from a depot for up to 12 weeks (Swanson et al., 2020b). As a further development of this technology, we developed a microsphere delivery platform which can be embedded into a tissue engineering scaffold. This approach combines the advantageous properties of EVs and their sustained release with a biomaterial scaffold optimized for bone regeneration (Swanson et al., 2020c). We demonstrated that this approach was sufficient to catalyze osseous wound healing of a calvarial defect *without* the transplantation of exogenous MSCs. Instead, we relied on released EVs to guide the fate of endogenous cells. We anticipate that these technologies are key to clinical translation of regenerative EV therapeutics. Other motifs of EV tethering, including ECM-inspired immobilization, covalent conjugation, and electrostatic interaction are described by Man et al. (2020).

Comparisons of MSC-based and EV-based regenerative technology consider that MSC sources are well-characterized and readily accessible (Moghadasi et al., 2021). While cell populations involved in tissue formation and repair are characterized for many tissues, ideal progenitor populations remain elusive for others or may not be suitable to autologous expansion and re-implantation. In these cases, EVs may be advantageous in that they can be produced at a larger scale than the cell source itself, and EVs from cell sources other than the target source may be able to catalyze regenerative outcomes. Since EVs can be stored for future use with relative ease and ability to be generated at small scales, EV-based regenerative therapeutics are further advantageous.

The potential implications of combined EV and biomaterial therapeutics allow for a tailored, predictable, tissue/patientspecific approach to regeneration, which is highly desirable by both patients and clinicians. EVs and biomaterial constructs are significantly easier to manufacture, store, and regulate compared to MSCs. These attributes represent significant cost savings, as well as increased likelihood of clinical adoption as these technologies would not require sophisticated technical expertise or equipment to implement into existing clinical workflows. As a result of the increased bio-instructive nature of optimized EV-biomaterial platforms, we believe that this may lead to simpler cell sourcing. EVs have been demonstrated to induce cell migration both *in vitro* and *in vivo*, sufficient to catalyze wound healing without requiring the transplantation of exogenous cells (Swanson et al., 2020b; Swanson et al., 2020c). In the same way, when exogenous cells are necessary, significant *ex vivo* autologous cell preparation (i.e., flow cytometry, *ex vivo* expansion) may be minimized as an instructive combination of EVs and biomaterial matrix provide sufficient selection criteria for regenerative cell populations, allowing more crude preparations.

## Conclusion

Predictability of regenerative outcomes is ultimate goal of next generation tissue engineering technology. In this Viewpoint, we highlight the synergy for development of biomaterial platforms which contain EVs, rather than rely on transplantation of stem cells. EVs in conjunction with tuned biomaterials matrices represent an exciting avenue for discovery, translation, and commercialization. We believe that EV-based biomaterial technologies hold the potential to democratize access to regenerative medicine therapeutics across medical disciplines and care settings given their decreased cost, increased manufacturing throughput, advantageous storage character and potentially easier point of care use. Successful clinical translation of these technologies will continue to rely on an intimate understanding of the molecular cargo encapsulated by EVs, interactions at cell-biomaterial interface and means of efficient EV delivery. We believe that regenerative potential represents a significant benefit to patients for a variety of conditions; therapeutic approaches which circumvent challenges associated with, such as EV-based therapies, will allow for more expedient clinical trials, regulatory approval, and widespread clinical adoption, ultimately improving patient care outcomes and quality of life.

## Figures and Tables

**TABLE 1:** Diverse demonstrations of various EV-based therapeutic applications selected from the literature

Regenerative target	Donor cell	Reference
Bone Mineralization	Bone Marrow MSCs	(Narayanan et al., 2016)
	Mineralizing Osteoblasts	(Cui et al., 2016)
	Osteoclasts	(Huynh et al., 2016)
	Adipose Derived MSCs	(An et al., 2019)
Bone Angiogenesis	Umbilical-cord Derived MSCs	(Zhang et al., 2012)
	Induced Pluripotent SC-derived MSCs	(Hu et al., 2015)
Intervertebral Disk Degeneration	Bone Marrow MSC, Nucleus Pulposus Cells, Adipose Stem Cells	(DiStefano et al., 2021)
Cardiac Ventricular Remodeling	C2C12 Myoblasts	(Yamaguchi et al., 2015)
	Cardiosphere-derived Cells	(Ibrahim et al., 2014)
Lung	Bone Marrow MSCs	(Lee et al., 2012)
Kidney	Bone Marrow MSCs	(Zhou et al., 2013)
Brain	Dendritic Cells	(Alvarez-Erviti et al., 2011)
Brain (Alzheimer’s Dz)	Murine Neuroblastoma Neuro2a Cells	(Yuyama et al., 2014)
Peripheral Nerve Repair	Adipose Derived MSCs	(Ching and Kingham, 2015)
	Schwann Cells	(Ching and Kingham, 2015)
Cutaneous Wound Healing	Epidermal SC	(Duan et al., 2020)
	Bone Marrow MSCs	(Ha et al., 2020)
Cartilage	Bone Marrow MSC	(Chen et al., 2018; Tan et al., 2021)
Gingival Mucosa	Gingival MSC	(Shi et al., 2017)

Note this is not an exhaustive list, many of these examples demonstrate *in vitro* or preliminary *in vivo* utility and serve as a basis for future investigation in the context of tissue engineering. Note this is not an exhaustive list, but aims to demonstrate breadth.

## Data Availability

Data sharing not applicable to this article as no datasets were generated or analyzed during the current study.
